# Supplementary material for: Autonomous Planetary Liquid Sampler (APLS) for In Situ Sample Acquisition and Handling from Liquid Environments
Source: Sensors (Basel). 2024 Sep 21;24(18):6107. doi: 10.3390/s24186107 (PMC11435854; doi:10.3390/s24186107)
Supplement: Supplementary file 1 [file sensors-24-06107-s001.zip › sensors-3181069-supplementary.pdf]

Supplementary Materials

Electronics Circuit Schematic

The schematic for electronics circuit and subsequent data push request to InfluxDB Cloud data bucket and visualisation using Grafana is shown in Figure S5. The connection between the relay terminals to the pump/valves/heater can be done as shown. Except the pump (Relay 3) and the heater mats (Relay 4) which operate with a 12 V DC source, all the valves are connected to the 24 V DC source (Relays 2, 5, 6, 7 and 8). The digital outputs from ESP32S3 are used to send the HIGH/LOW signal to the 8-Channel Relay module and the analog outputs from the 10 K NTC Thermistor (T) and the Hall effect flow sensor (F) are connected as analog inputs. The ESP32S3 does the job of connecting to the WiFi and pushing the data into the desired InfluxDB Cloud data bucket. Note that the data bucket and access token must be created beforehand. Once the data is in the bucket, they can be verified using the inbuilt "SQL Synx" option. Post this, a visualisation dashboard can be created in Grafana by selecting the specified data bucket as the data source.

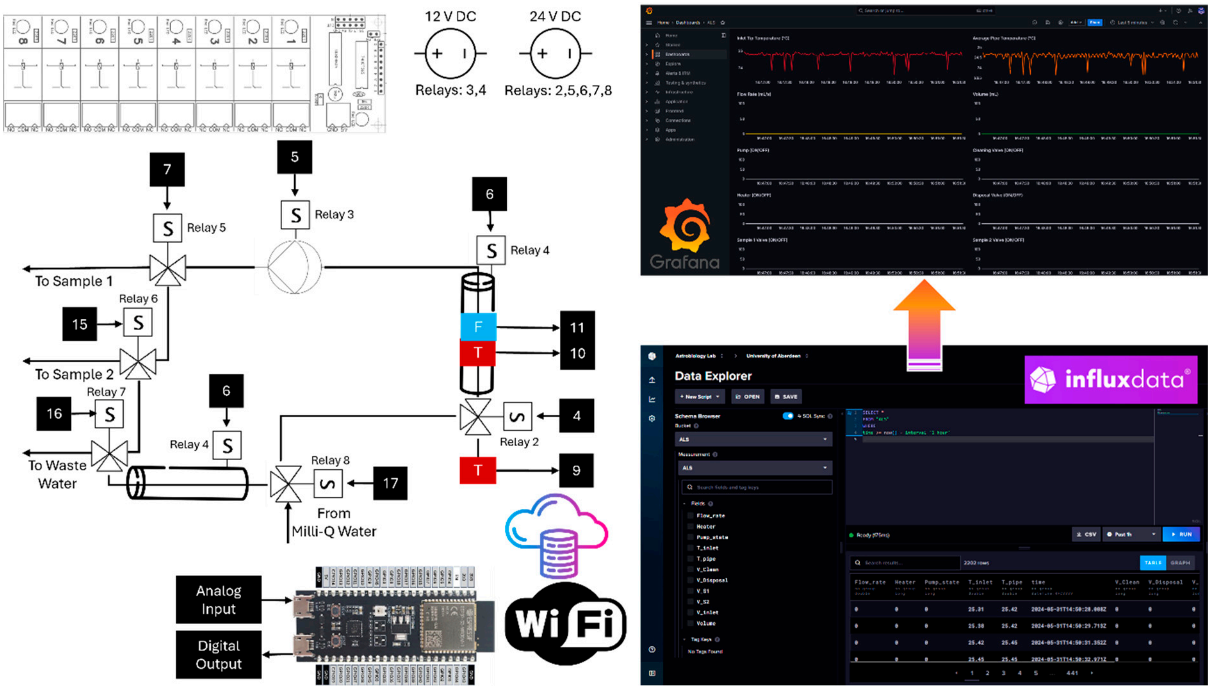

Figure S1. Electronics Circuit Schematic of the APLS proof-of-concept prototype

Source Code

All the source code for uploading to the ESP32S3 developed board and Simulink model and results can be found at https://github.com/miracleisraelnazarious/APLS.

Grafana Plot Query

The below script in Figure S6 was used to visualise the data from the InfluxDB Cloud data bucket named "ALS". Note that, this script must be used individually for each visualisation window by changing the "VARIABLE_NAME" to be displayed. The user

can choose to aggregate the data points in the field mentioned “XXX” with a time parameter. E.g., 1s (second), 1m (minute), 1h (hour), 1d (day), etc.

```
from(bucket: "ALS")                        // —— Source
  |> range(start: -1h)                     // —— Filter on time
  |> filter(fn: (r) => r._field == "VARIABLE_NAME") // —— Filter on variable
  // |> aggregateWindow(every: “XXX”, fn: mean)     // —— Averaged values
```

Figure S2. Sample Grafana plot query
